# Study Protocol of the Parents in Child Nutrition Informing Community (PICNIC) Peer Education Cohort Study to Improve Child Feeding and Dietary Intake of Children Aged Six Months to Three Years Old

**DOI:** 10.3390/children7010003

**Published:** 2019-12-27

**Authors:** Richard Ball, Rebecca Vaschak, Andrew Bailey, Gail Whiteford, Tracy L. Burrows, Kerith Duncanson, Clare E. Collins

**Affiliations:** 1 Mid North Coast Local Health District, Port Macquarie, NSW 2444, Australia; Andrew.Bailey@health.nsw.gov.au (A.B.); gwhiteford@csu.edu.au (G.W.); 2 Nutrition and Dietetics, School of Health Sciences, Faculty of Health, The University of Newcastle, University Drive, Callaghan, NSW 2308, Australia; tracy.burrows@newcastle.edu.au (T.L.B.); clare.collins@newcastle.edu.au (C.E.C.); 3 Coffs Harbour Women’s Health Centre, Coffs Harbour, NSW 2450, Australia; rvaschak@genhealth.org.au; 4 School of Community Health, Charles Sturt University, Port Macquarie, NSW 2444, Australia; 5 Priority Research Centre in Physical Activity and Nutrition, The University of Newcastle, Callaghan, NSW 2308, Australia; kerith.duncanson@newcastle.edu.au; 6 Health Education and Training Institute (HETI), Gladesville, NSW 2111, Australia

**Keywords:** infant, child, feeding, nutrition, peer education, social media

## Abstract

One in five Australian pre-schoolers are overweight or obese, meaning the first years of life are vital for obesity primary prevention. Parent child feeding practices impact on children’s dietary intake, which in turn impacts on their weight status. Parents’ child feeding beliefs are heavily influenced by parenting peers. The aim of this cohort study is to evaluate the impact of the Parents in Child Nutrition Informing Community (PICNIC) study on parents feeding practices and diet quality. The secondary outcomes are the perceptions of trained peer educators and education recipients based on their involvement in PICNIC. One hundred parents with a child aged 0–2 years at time of recruitment will participate in peer educator training, then disseminate nutrition and child feeding content to other parents over an intervention period of 12 months, supported by project-specific, evidence-based social media pages and website. An additional 100 new parents, recruited by peer educators, will participate in the study as nutrition education recipients. Both peer educators and education recipients will complete quantitative child feeding surveys before and during the 12 month intervention and a dietary intake survey at a time point 12 months post intervention. Following the intervention, 30 education recipients will be asked to participate in semi-structured phone interviews about their experiences with PICNIC. Peer educators will contribute as co-researchers and active participants in the evolution of the PICNIC model. This study will contribute to enhanced understanding of contemporary health literacy strategies for communicating nutrition and feeding messages to new parents and the impact of these strategies on parents feeding practices and children’s dietary intake in a community setting.

## 1. Introduction

Overweight, obesity and associated chronic disease pose ongoing, unsustainable and escalating burden on the public health system, with an estimated annual public health cost to Australia in 2011–12 of $8.6 billion [1]. Overweight and obesity rates in Australia increase consistently with age, from 20% of pre-schoolers to 63% of adults [2]. Half of Australia’s healthy weight children are predicted to become overweight or obese adults [3]. Interventions aimed at influencing overweight and obesity therefore need to target the underlying causes of the development of obesity-related behaviours.

Dietary patterns and other eating behaviours develop in childhood and track throughout a person’s life, contributing to their relative risk of preventable disease [4]. The diets of Australian children deviate from the dietary guidelines from an early age, with children aged two to three years consuming more than double the maximum recommended intake of energy from discretionary food and less than half their recommended serves of vegetables [3]. Factors that contribute to this deviation from the dietary guidelines include parental feeding practices, which impact on diet quality, food quantity and weight gain in infants as young as six months old [5]. The early years of life are therefore vital in influencing the development of health-promoting dietary patterns and food related behaviours associated with the risk of lifestyle-related disease [6].

The development of children’s early life eating patterns, taste preferences and dietary intake, behaviours and attitudes towards food are largely determined by parents and carers [7] and continue to be determined in this manner until adolescence [5]. The feeding practices of parents and carers can enhance or hinder children’s healthy eating development [8,9,10]. Once established, child feeding practices and eating habits are difficult to change [11]. Parents report that child feeding is one of the most challenging aspects of parenting and is a leading cause of anxiety amongst parents and children [12,13]. Parents desire good health for their children but early intentions about healthy feeding and eating can be thwarted by difficulty translating available information into practice and the demands of implementing behaviour management techniques in an increasingly obesogenic food environment [14].

Child feeding practices are the behaviours associated with food provision to children. They strongly predict the quality and quantity of foods consumed and underpin lifelong eating patterns [15]. Feeding practices that are associated with negative health consequences include coercion to eat, frequent offering of food, using food as comfort or reward and inappropriate food restriction or exposure to food. In the short-term, these strategies may temporarily appease a child but in the longer term can produce undesired outcomes such as poor self-regulation of intake, eating in the absence of hunger, rejection of healthful foods, preference for energy-dense, nutrient-poor foods, food anxiety and avoidance [5,15,16,17,18,19]. Feeding practices are intertwined with culture, tradition and parenting style as well as health, so influencing or changing parents’ child feeding beliefs, attitudes and perceptions is complex [15]. Providing a balanced, healthy diet to children is challenging in the context of a contemporary food environment, which facilitates overconsumption and offers frequent opportunities to consume energy-dense, nutrient-poor foods [20]. Nutrition interventions aimed at improving the dietary intake of infants and toddlers therefore need to include behavioural strategies that parents can use to convert their healthy child feeding intentions into practice.

New parents may have limited exposure to evidence-based nutrition and feeding information and be exposed to non-credible information in critical transitional periods in children’s growth, development and formulation of food preferences [21]. The prevailing model of health care delivery remains centre-based child and family health care in a community health setting. Feeding information received in Child and Family Health Service visits provide early support but may not be sufficient in dosage, individualisation, specificity or timed appropriately to influence sustained change [22]. Additionally, parents are unlikely to associate their infant or child’s dietary intake with longer term health outcomes, or to prioritise child feeding highly enough to seek assistance [22].

Friends, family, the internet and smartphone applications (apps) are reported to be parents’ most regular source of child feeding and nutrition information [23]. Ninety five percent of Australians aged 18–34 years, the age when most individuals become new parents, own a smart phone mobile with internet capacity [24]. Easy access to the internet and apps exposes parents to diverse sources of child feeding information, which can create confusion for well-intentioned parents [21]. Trustworthy online sites and content are therefore critical in enabling effective use of these sources for as a health information source for parents [23]. Social media can offer a social interaction mechanism for both individuals and communities [25]. It shows promise as a tool for peer-to-peer education and has been used by parents to access accurate infant feeding advice from other parents [21,26]. More research is required to understand and assess the effect of social media as a health communication tool [25].

In a health context, peer education is an approach where community members provide information on health behaviours attitudes, values and behaviours to others of a similar age or experience, or family life cycle stage, on behalf of health professionals [27]. Peer education has been shown to be effective for a range of health behaviour messages in various settings [28,29,30,31,32,33]. Although interventions targeting nutrition and feeding practices are typically modelled on traditional dichotomies of ‘health professional’ and ‘patient’, diet-related behaviours are more likely to change if educators share similar demographics and nutritional concerns [34]. This is explained by the principles of the Theory of Planned Behaviour, whereby behaviour is determined by a complex interaction between attitude, perceived control, normative beliefs, motivation to comply with norms, and the interactions between these factors [14].

New parents frequently form groups and long-lasting social connections with other parents who have infants of a similar age [34]. Such groups, often initiated by child and family health nurses, potentially offer a cost-effective, population level system for sharing of evidenced-based nutrition information, The timing and format of peer parent groups have the potential to maximize uptake and provide a sufficient message dose for sustainable health behaviour modification, as a group’s social support and norms are resistant to change [33]. Peer education is particularly suited to rural implementation, addressing social isolation [35] by negating the need for rural new parents to travel to access health services while creating a sense of connection within a community. Parents report a willingness to seek evidence based nutrition education for their own purposes and undertake two or more hours of training in view of on training their peers [23].

This study protocol describes the methods for implementing and assessing the effectiveness and end user acceptability of PICNIC, a peer educator model with online and social media components, for engaging parents of young children and influencing the social norms around nutrition and child feeding practices.

### Study Aims

**Figure 1:**
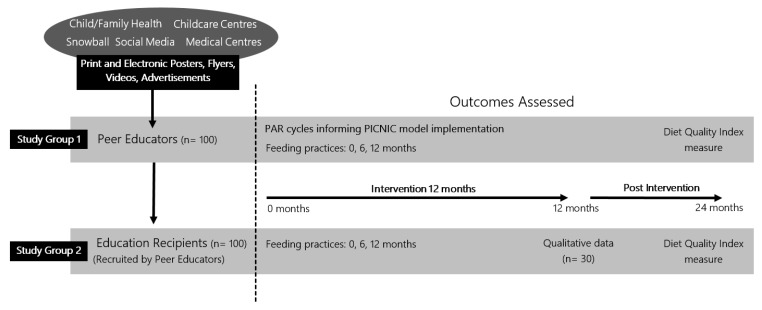
Parents in Child Nutrition Informing Community (PICNIC) study design and recruitment.

The primary aim of the PICNIC study is to assess the effect of a peer education model supported by a website and social media on infant/child diet quality and their parents’ feeding practices. The secondary aim is to describe the perceptions of primary caregivers who have participated in the study as a peer educator or had their role in infant feeding supported by a peer education model with face-to-face, website and social media components. Figure 1 provides an overall summary of this study.

The hypotheses are that infant and child feeding and nutrition information, modelled and delivered by peer educators over 12 months will:

- Improve the infant/child feeding practices of parents from baseline;
- Improve their infant/child’s diet quality against population norms;
- Improve self-reported child feeding experiences of parents; and

At an implementation level, this study will seek to examine if and how a peer, education approach can improve the PICNIC study implementation model in line with the principles of research co-design.

## 2. Materials and Methods

### 2.1. Ethics Approval

Approval for the study was obtained from North Coast New South Wales Human Research Ethics Committee HREC Ref Number: LNR179 (4/12/17).

### 2.2. Intervention Development

The PICNIC model has been informed by The Food For Kids Mid North Coast (FFKMNC) pilot study [21]. The FFKMNC study reported the experiences of new parents (n = 28) in the role of nutrition peer educators and demonstrated peer to peer education is acceptable with new parents to share evidence based infant nutrition and child feeding messages [21]. Recommendations from the FFKMNC that informed the PICNIC model included recruitment earlier in the parenting cycle (before child aged six months), focusing information on feeding practices which positively influence feeding behaviour, improved access to online content via social media and the creation of a project specific website developed by an established Health Promotion Team within a Health Service framework [21].

### 2.3. Study Design

PICNIC is a mixed methods cohort study underpinned by Participatory Action Research (PAR) Methodology [36]. The PAR methodology is defined by engaging participants as partners in research and is based on reflection, data collection and action. The participants recruited for this study will be categorised in two groups: (1) Peer Educators and (2) Education Recipients, for which the project role, qualitative data collected and the recruitment process will differ (Figure 1).

Peer educators will work collaboratively with the research team to further guide ongoing intervention characteristics in a PAR approach. This will ensure modification and adaptation of the project model in partnership, consideration of participant and group characteristics and futureproofing with regards to shifting trends and evolving technologies.

### 2.4. Recruitment/Participants

The PICNIC study will use a progressive recruitment strategy over a one-year period from December 2019 to December 2020. Peer educators will be recruited into study group 1. Peer educators will be responsible for the recruitment of education recipients into study group 2. This group will consist of other parents within their social networks with whom peer educators are likely to share information over the intervention period. Different recruitment processes (Figure 1) will be used to recruit the two study groups separately. We aim for nine peer educators and nine education recipients to be recruited per month with all recruitment ceasing December 2020. Each peer educator and the education recipients they recruit will undertake the same intervention timeline (i.e., 24 months from the date of their peer educator workshop).

### 2.5. Study Group 1 Recruitment: Peer Educators

Peer educators will be recruited at Mid North Coast Local Health District (MNCLHD) Child and Family Health services, Early Childhood services, Medical services, and through social media and other parent/carer specific programs. Recruitment will be enabled through existing partnerships between recruitment sites, community organisations, research and health service teams. Additionally, snowball sampling will be encouraged, with consenting parents promoting participation to their partners and other parents. To mitigate the risk of selection bias, it will be explained to recruitment agencies that eligible parents from all social and demographic groups are invited, with a broad representation desirable.

Print and electronic posters, flyers, advertisements and videos with recruitment contact details will be provided to recruitment sites to display in facilities, distribute to potential participants and to disseminate via existing program and site social media pages. Child and Family Health services, Childcare services, Medical services will provide recruitment packs consisting of information and consent forms to potential participants or direct them to the ‘expression of interest’ web page on the project specific website. Electronic Facebook recruitment advertisements will direct interested parents to the ‘expression of interest’ page on the project website for additional program information and consent forms. The content and literacy level of all recruitment materials will intentionally target parents from across a range of sociodemographic groups with the aim of further mitigating selection bias. Consenting peer educators will submit signed consent forms prior to attending a peer educator training workshop.

### 2.6. Study Group 2 Recruitment: Education Recipients

Peer educators will be provided with study group 2 consent packs and recruitment guidance at the peer educator training workshops. Each pack will include a consent form and a participant information statement describing the study. Peer educators will be asked to recruit at least (but not limited to) one other new parent from within their social network who has(ve) an infant/child aged 0–2 years within a month of the peer educator workshop. Peer Educators will provide all parents within their social network who meet the inclusion criteria, the opportunity to become a formal education recipient and contribute data to the PICNIC project.

### 2.7. Inclusion and Exclusion Criteria

Peer Educators: Parents and primary carers of an infant aged from birth to two years, are aged 18 years and older, live within the MNCLHD area and are able to understand written and spoken English. Either or both parents can participate as peer educators.

Education Recipients: Parents and primary carers of an infant aged from birth to two years, are aged 18 years and older, live within the MNCLHD area and are able to understand written and spoken English. Education recipients will have social contact and be exposed to information and resources provided by a peer educator. Recipients do not need to have computer access or use social media to be included.

Exclusion criteria (Peer Educators and Education Recipients: Parents and primary carers with only children whose nutrition/feeding are influenced by a medical condition, speech pathology, disability or who are unable to understand written and spoken English.

### 2.8. Intervention

The intervention will have two arms: Arm one is peer educator training in infant/child nutrition and feeding and arm two is peer educators on-sharing infant/child nutrition and feeding information to other new parents (education recipients). Study arm one will include a single two hours face-to-face workshop, ongoing circulation of resources, training via social media and information requests by peer educators over the 12 months intervention period. The 12-month intervention period will commence for each participant on the date they attend the initial face to face workshop and will conclude the same date 12 months later. For each participant in either arm one or two the overall study period will be 24 months, a 12-month intervention and a data collection point 12 months after the intervention has concluded. All training and ongoing support will be facilitated by the PICNIC researchers.

**Figure 2:**
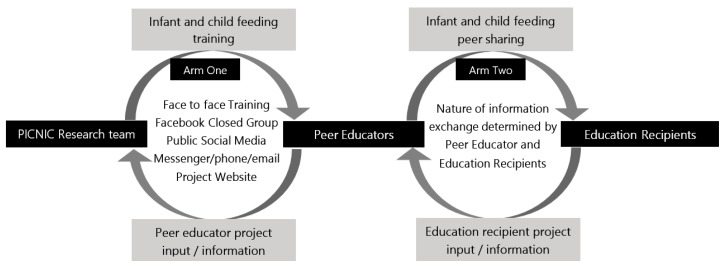
Infant/child nutrition and feeding information exchange in the PICNIC study.

Study arm two will commence for education recipients approximately when the peer educator that were recruited by attends the initial face-to-face workshop. Peer educators will determine all aspects of information on-sharing including content, frequency and method by which information is disseminated, based on the needs and requests of other parents and parent groups. Peer educators will be provided with weekly reminders (via a closed social media group) to provide feedback to the research team and make suggestions that will inform content, process and other program components throughout the intervention period. This data from peer educators will be collated and actioned as outlined in Figure 2.

### 2.9. Intervention Content

Health education information to be provided to peer educators in the PICNIC study has been informed by participants in the FFKMNC study [21] and developed by Accredited Practising Dietitians specialised in paediatric feeding for healthy growth and development. Consistent with a PAR approach, peer educators will work collaboratively with the research team over the intervention period, to moderate, shape and guide content, to ensure information is suitably tailored for themselves and peer groups.

Feeding messages and resources will be based on evidence-based principles [5,15,16,17,18,19] and grouped by nutrition and child feeding topic area that are relevant to infants approximately six months of age and onwards. Although breastfeeding is a parallel infant feeding issue for this demographic, it is not the primary focus of this study. Additionally, message content will address the components of the Theory of Planned Behaviour in an effort to impact actual food-related behaviour. These consist of normative beliefs, subjective norms, behavioural beliefs and attitudes, control beliefs, perceived behavioural control and behavioural intent specific to nutrition and child feeding practices [14].

The core nutrition and feeding topics that will underpin the PICNIC content are: Introduction of Solids, Responsive Feeding, Australian Guide to Healthy Eating, Food Exposure, Division of Responsibility, Food Rewards, Pressure to eat, Food Restriction, Family Meals, Role Modelling, Common Feeding Concerns and Healthy Growth (Supplementary Table S1). These topics were determined by participants in the FFKMNC study [21] and the research team, specialists in infant/child feeding. Topics will evolve in response to the PAR component of the PICNIC study.

### 2.10. Peer Educator Training Workshops

Three two-hour face-to-face workshops per month will be conducted at Community Health centres located within the MNCLHD area, with frequency at each location dependent on recruitment numbers. Each prospective peer educator will attend one workshop and those who consent to PICNIC participation will become a ‘PICNIC peer educator’. The workshops will contain intervention content and logistic information covering the roles, responsibilities and activities associated with being a ‘PICNIC peer educator’. The nutrition and feeding content will be delivered by Accredited Practising Dietitians and will be tailored to the age and developmental stages of participant’s children. Supplementary content on the PICNIC website will be referred to throughout the workshop to increase peer educators’ familiarity with the nutrition and feeding content and increase their confidence in navigating the PICNIC site post workshop.

### 2.11. Peer Educator Support and Resources

Peer educators will be provided with ongoing training, support and resources to share throughout the following 12-months. To protect the integrity of evidence-based nutrition information, the intervention content will be located on a website developed specifically for the study (picnicproject.com.au) following consultation with local parents and past participants of the FFKMNC study [21]. The information on the website will be organised by age group (6–12 months, 12–24 months and 24–36 months) to reflect the stages of child feeding, nutritional needs of young children and assist website navigation by parents. Feeding messages provided to peer educators via social media will address key content areas to capture interest and will refer back to the website for participants to read more about a particular subject.

At each PICNIC workshop, peer educators will be invited to join the Facebook closed group “PICNIC Peer Educators”. Peer educators will be provided a group invitation via Facebook, with requests accepted by the research team (RB, BV) group administrators. The closed group will function as a location for continued education, resource provision, house social media posts and a communication vehicle between peer educators and the research team. Regular intervention messages, website referrals and education sessions will be posted, and it is anticipated this page will house discussion on feeding topics between peer educators and the research team. Public social media pages (Facebook: PICNIC Mid North Coast, Instagram: Picnic mnc) will be created to provide peer educators the ability to easily share posts with education recipients, contribute to program awareness and to recruit peer educators to the study. Peer educators will be encouraged to ‘like’ and follow these sites along with other parents within their social groups.

### 2.12. Social Media Posts/Messages

**Figure 3:**
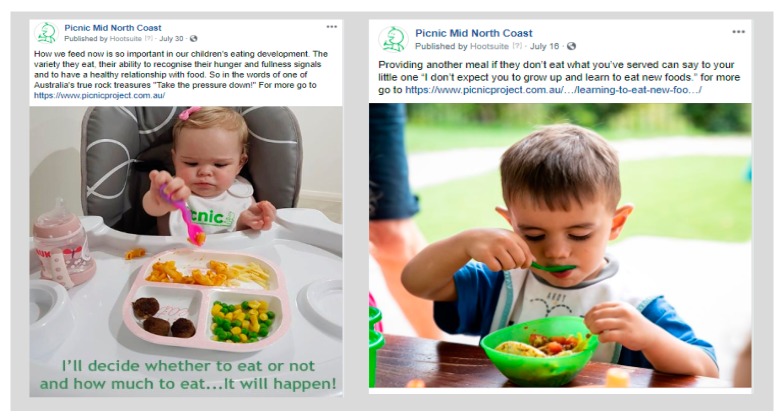
Examples of proposed “meme style” social media messages on PICNIC Mid North Coast.

Messages targeting intervention content will be formatted into short “meme style” sharable posts suitable for Facebook, Instagram and other online platforms (Figure 3). The structure and format will vary from video images to text and links to the PICNIC and other sites with consistent content and messaging. To enhance a ‘local’ and ‘real’ feel, imagery and video of participants and other local parents and their infants in local settings will be used (with consent) where possible. Posts that are created will be mapped to specific intended outcomes, such as guiding parents to website content, increasing engagement and encouraging discussion and interaction. Message posts will be created by the research team or re-purposed from other similar Facebook sites, with acknowledgement where appropriate. Facebook post content and format will be determined over the 12-month intervention period based on ‘post’ performance and peer educator input. The terms of use governing all project content was developed in partnership with the MNCLHD ensuring consistency with NSW Health code of conduct and media policy.

Each message will be posted simultaneously on the Facebook closed group (for peer educators’ education and awareness) and onto the public social media pages for ease of sharing by peer educators. Approximately five to seven feeding messages per week will be posted at times of day when page followers are most likely to be online. Information about the usual social media use habits of education recipients will be obtained from peer educators over the intervention period.

It is anticipated the majority of participants will use Facebook or Instagram. Alternative dissemination methods (as determined by peer educators) will be made for those who choose not to use social media, ensuring an adequate dosage of intervention messages, education materials and feeding discussion. The utilization of each method will be monitored to determine the demand for alternative social media platforms or other communication strategies in the future.

### 2.13. Peer Educators and Education Recipients Information Exchange

The role of the peer educator will be dissemination of nutrition and feeding information to others within their social network who have an infant in the targeted age range. After attending the workshop peer educators will be asked to identify themselves as having received evidence-based ’PICNIC’ training and be accessible for a social group’s nutrition and feeding information requirements and to share nutrition and child feeding information within their social networks. Peer educators will engage with education recipients either individually or from within their own networks over the intervention period.

Peer educators will be provided suggestions and examples of information sharing in the face-to-face training workshop and throughout the intervention period based on experiences of other peer educators and participants in the pilot FFKMNC study. The nature of information exchanges will be determined by each peer educators and the requirements of their social network within the limits of the pre-determined general nutrition and feeding intervention content.

### 2.14. Data Collection

Baseline surveys will be completed by peer educators in the training workshop and by education recipients after consenting to participate in the study. These surveys will be completed via links to the online demographic information and feeding practice questionnaires on the project website picnicproject.com.au. The research team will be available to assist education recipients to complete baseline questionnaires if required. Peer educators and education recipients will complete demographic data once and feeding practice surveys at three time points over the intervention, at baseline, six months and 12 months post-workshop. Peer educators and education recipients will be reminded to complete data with three text messages reminders only, within the due month. The age of the infant/child at each data collection time point will determine which surveys need to be completed by participants. Print copies of feeding practices and demographic questionnaire will be available to participants if preferred. A link to the Healthy Eating Quiz [37] will be sent to participants 12 months after the intervention has concluded. Data collection methods may be modified based on feedback from peer educators and completion rates, with details of any iterative modifications recorded. The impact of social support as part of the peer education role (along with other components such as intervention exposure) on dietary intake and child feeding will be assessed, but the social support component between peer educators and education recipients will not be able to be measured specifically.

### 2.15. Demographic Information

Parent age, gender, infant/child age, spoken language at home, indigenous status, education level, employment status and number of children will be collected in the demographic questionnaire for each participant. Intervention-related information including interaction with parenting networks and initial preferred communication methods will be collected and accounted for in analysis.

### 2.16. Parent Feeding Practices

Peer educators and education recipients will complete surveys at baseline, six and 12 months to identify whether the intervention has positively changed parents’ child feeding practices. The behaviours to be measured directly relate to the intervention content and mediate both short and long term dietary and feeding behaviour outcomes [5,15,16,17,18,19]. The Feeding Practices and Structure Questionnaire Milk and Solids Version and the restriction construct from the Feeding Practices and Structure Questionnaire (FPSQ-28) [38,39] will be available using the Survey Monkey^tm^ platform, take approximately 10 minutes to complete and measure change within each feeding construct over time.

Change in child feeding as measured by mean (SD) for the constructs of: Demand vs. routine (4–5 items, total possible score 20–25); Sit down meals (4 items, total possible score 65); Meal environment (6 items, total possible score 30); Trust in feeding (13 items, total possible score 65); Pressure to eat (7–11 items, total possible score 35–55); Food to calm (5–6 items, total possible score 25–30); Reward for eating (4 items, total possible score 20); Reward for behaviour (11 items, total possible score 55); Overt Restriction (4 items, total possible score 20); Covert Restriction (4 items, total possible score 20) will be analysed over time (Table 1).

### 2.17. The Australian Recommended Food Scores for Pre-Schoolers (ARFS-P)

Peer educators and education recipients will be sent a link to complete an online survey “The Healthy Eating Quiz” [37] 12 months after their completion of the intervention period. The Healthy Eating Quiz survey takes approximately 10 minutes to complete and is able to generate The Australian Recommended Food Scores for Pre-schoolers (ARFS-P) [37]. The ARFS-P is a paediatric diet quality index measure validated for use in children aged two to five years to assess usual diet quality, food variety and the nutritional adequacy of dietary intakes of Australian pre-schoolers.

### 2.18. Participatory Action Research (PAR)

Peer Educators will be engaged as co-researchers to work collaboratively in the evolution and modification of the PICNIC model and to illuminate the lived experiences, perceptions and value placed by new parents in their role as a peer educator. Data will be collected from a variety of sources including document analysis of correspondence, field notes, analysis of comments and correspondence of social media and individual or group interviews. Individual and group interviews with peer educators will be conducted by an Associate Investigator (GW), an expert in qualitative data collection and unknown to participants, at two to three time points over the intervention period, to capture participants input at different stages of their program involvement. Feedback on all website, social media content and other program aspects will be sought from peer educators, reflected on and actioned on a two-monthly cycle over each participants’ intervention period. Outcomes from each PAR cycle will be documented, used in the PICNIC study co-design implementation and analysed to further to determine perspectives of peer educators’ experiences in the PICNIC project.

### 2.19. Qualitative Inquiry Data Collection

Peer education recipients who have had 12 months exposure to the PICNIC intervention will be invited to participate in semi-structured phone interviews to discuss their experiences of being supported by the PICNIC model and their perceptions of their child-feeding efficacy. Consenting participants will be contacted to organise a mutually acceptable time for the interview, which will be conducted by associate investigator (GW). The interview guide will be co-designed by the research team and peer educators and field tested on peer educators. Modifications will be made by consensus, based on feedback from peer educators.

Interview telephone calls will be initiated from a Community Health Centre, with interviewees answering the call from the naturalistic setting of their own home. Education recipients will be asked approximately eight to ten questions exploring their infant feeding experiences being supported by the PICNIC model and their perceptions of their child-feeding efficacy. The interviews will be audio recorded using a digital recorder. Phone interviews are anticipated to take between 30 and 45 minutes.

The digital audio recordings will be sent electronically to a transcription service (Pacific Transcription) in a de-identified format. The transcription service will transcribe each interview verbatim and return it electronically to the researcher in a word document format. Transcripts will be re-identified so participants can be invited to review transcripts for accuracy prior to analysis, amended if necessary, and then de-identified. Interview transcripts will be checked for accuracy against recordings prior to thematic analysis.

### 2.20. Primary Outcomes

1. Improved child feeding as measured by mean (SD) for the constructs of: Demand vs. routine; structured meal timing; sit down meals; family meal environment; role modelling; division of responsibility/trust; pressure to eat; food to calm; food rewards; (FPSQ milk/solids); restriction (FPSQ-28) over time and compared to reference populations/samples.
2. Dietary intake as measured by core food variety, overall diet quality score from ARFS-P in comparison to normative population data.

### 2.21. Secondary Outcomes

1. Parents and primary carers experiences as peer educators in the PICNIC project.
2. Parents and primary carers infant feeding experiences of being supported by the PICNIC model and their perceptions of their child-feeding efficacy.

### 2.22. Data Analysis

Demographic data, feeding practices and child dietary intake data will be collected online and exported as Excel files. Following data cleaning and de-identification, files will be uploaded into STATA by one researcher (RB). Normality checks on all data will be conducted prior to further analysis and no imputation of missing values will be carried out. Descriptive statistics will be reported as mean (SD) or median (interquartile range), depending on whether or not data are normally distributed.

Changes in child feeding over time will be assessed measured by mean (SD) for the constructs of: Demand vs. routine; structured meal timing; Sit down meals; Family meal environment; Role Modelling; Division of responsibility/Trust; Pressure to eat; Food to calm; Food rewards; Restriction (Overt/Covert). Mean values for each domain will be compared over time and changes over time analysed at an individual level using appropriate interpretive statistical tests, and accounting for confounding factors where possible.

Dietary intake analysis will include the following measures: Core food variety, overall diet quality score. These will be measured against age-matched controls at 24 months post intervention using the validated Healthy Eating Quiz [37]. Mean values for all variables will be compared using appropriate comparative statistical tests. Statistical analysis will be completed using STATA statistical software (Version 10, College Station, TX, USA).

A sample size of at least 100 peer educators and 100 education recipients will be feasible for analysis of the PICNIC study within existing resources, allowing for comparison with historical data [40] age and population matched controls and to be adequate to detect a difference in dietary intake between groups. We have conducted a power and sample size calculation as follows: 1 sample test with 80% power at 5% significance level and to detect a 4 points difference (greater or less) in ARFS (diet quality) between the historical control (ARFS = 36) we need to conduct 60 ARFS surveys of 3-years old. To detect a 2 points difference, n = 237, or for a 3-point difference, n = 106. Therefore, we will aim to conduct at least 200 ARFS surveys, which will allow for both detection of a 2 points difference and also to allow for potential stratification to be accounted for in analysis.

The experiences of parents and primary carers in their role as peer educators and having their infant feeding supported by the PICNIC program will be analysed. First level analysis will consist of preliminary coding of transcripts by two researchers (GW, RB), each working independently. This will be followed by discussion and consensus around codes created in first level analysis. A descriptive summary of each resulting theme will then be collated, with supporting quotes added to provide context and evidence for the theme. The thematic analysis of the qualitative component of the study will be prepared for publication, as will the participatory action outcomes.

This study has been submitted for registration with Australian New Zealand Clinical Trials Registry (ANZCTR): Record No. 378259.

## 3. Discussions

Evaluating the translation of nutrition research into practice is currently an implementation priority in population focused health services. It is critical to evaluate whether early childhood-focused nutrition education and skill development approaches meet the needs of parents and whether this leads to changes in child feeding practices and indicators of child dietary intake. The PICNIC study will pioneer research using the peer nutrition educator model to determine the impact of a population level intervention on parent’s child feeding practices and child diet quality among rural children over a 12-month timeframe, and in comparison to national level dietary survey data.

The PICNIC study will extend on current research into early childhood nutrition by (i) examining the child feeding practices of parents in a community setting, using a peer education intervention model that could be applied at a population level (ii) including collection of both dietary intake and child feeding information of children aged between six months old and three years old over a 24-month period, thereby allowing assessment of medium term intervention effectiveness; (iii) a qualitative component to complement, extend on and provide context to quantitative data and associated implementation factors and (iv) investigating nutrition-related factors in a representative sample of rural children aged six months to three years, a demographic group previously under-represented in childhood nutrition studies.

This study will build on previous research into the role of parents in children’s dietary intake. Although studies in Australia and the USA have examined the impact of intensive nutrition interventions on the dietary intake of toddlers and primary school aged children, there has been limited study of outcomes from peer nutrition education models, particularly at a population level and particularly those living in rural areas. Dietary intake of children aged six months to two years is not widely available, particularly for children in a free-living community setting. Similarly, the child feeding practices of parents with children in this age group are not commonly reported in the literature. This is substantiated by the relative absence of validated tools for assessing parental child feeding practices of infants aged six to 36 months. The application of an existing validated tool for child feeding practices in a younger age cohort provides further support for the potential of PICNIC.

The community-based, cohort study design may result in some selection and information bias in this study, however these will be mitigated by the inclusive recruitment strategy, and by collection, analysis and reporting of demographic characteristics, and of dissemination and uptake of educational content.

Ongoing implementation of the PICNIC study, embedded within local health service delivery will allow for ongoing quality improvement, based on quantitative and qualitative research findings. With additional ethics approvals, further research will be feasible as children get older. This will facilitate a longitudinal study that will make a unique contribution to the understanding of child feeding and dietary intake in Australia.

## Figures and Tables

**Table 1:** PICNIC study feeding practice constructs and age range. FSPQ: Feeding Practices and Structure Questionnaire.

Age Range (Months)	0–6	6–12	12–24	24–36
FPSQ Version	Milk	Solids	Solids + Rewards	Solids + Restriction
Demand vs. routine	4 items	5 items	5 items	5 items
Sit down meal		4 items	4 items	4 items
Meal environment		6 items	6 items	6 items
Trust	13 items	13 items	13 items	13 items
Pressure to eat	7 items	11 items	11 items	11 items
Food to calm	5 items	6 items	6 items	6 items
Reward for eating			4 items	4 items
Reward for behaviour			11 items	11 items
Overt Food Restriction				4 items
Covert Food Restriction				4 items

## Supplementary Materials

The following are available online at https://www.mdpi.com/2227-9067/7/1/3/s1, Table S1: PICNIC Intervention Content.
