# Supplementary material for: Study Protocol of the Parents in Child Nutrition Informing Community (PICNIC) Peer Education Cohort Study to Improve Child Feeding and Dietary Intake of Children Aged Six Months to Three Years Old
Source: Children (Basel). 2019 Dec 27;7(1):3. doi: 10.3390/children7010003 (PMC7023363; doi:10.3390/children7010003)
Supplement: Supplementary file 1 [file children-07-00003-s001.pdf]

Supplementary Table S1: PICNIC Intervention Content

Introduction of solids<br>- Signs your baby is ready for solid food<br>- Solids from 6 months<br>- Iron Rich foods<br>- Food texture<br>- Choking risk<br>- Food safety	Food Restriction<br>- Restricting amount of food<br>- Prevalence of restricting amount eating<br>- Impact of disrupted appetite<br>- Management of non-core foods<br>- Overt Restriction<br>- Covert Restriction
Responsive feeding<br>- Signs my baby is hungry or full<br>- Food to Calm	Australian Guide to Health Eating<br>- Age appropriate nutrition principals<br>- Core/ Non - Core foods
Food Exposure<br>- New food phobia<br>- Food rejection by infants<br>- Neutral exposure<br>- Variation between infant/children taste<br>- Food Sensitisation	Meal Structure<br>- Family meals<br>- Meal Environment<br>- Mess at meal times<br>- Distractions
Division of Responsibility<br>- Parent provide child decide<br>- Trust in roles	Role Modelling<br>- Role modelling and learning to eat<br>- Impact on neophobia<br>- Positive and negative role modelling
Food Rewards<br>- Food reward for eating<br>- Food reward for behaviour<br>- Strategies for food reward environment	Common Feeding concerns<br>- Problem feeding<br>- Picky v problem eaters
Pressure to eat<br>- Prevalence of pressured eating<br>- Short long term outcomes of pressured eating<br>- Examples of pressure<br>- Strategies navigating food environment	
